# Identification of genomic sites for CRISPR/Cas9-based genome editing in the *Vitis vinifera* genome

**DOI:** 10.1186/s12870-016-0787-3

**Published:** 2016-04-21

**Authors:** Yi Wang, Xianju Liu, Chong Ren, Gan-Yuan Zhong, Long Yang, Shaohua Li, Zhenchang Liang

**Affiliations:** Beijing Key Laboratory of Grape Science and Enology and Key Laboratory of Plant Resource, Institute of Botany, Chinese Academy of Sciences, Beijing, 100093 P.R. China; University of Chinese Academy of Sciences, Beijing, 100049 P.R. China; USDA-ARS Grape Genetics Research Unit, Geneva, NY 14456 USA; College of Plant Protection, Shandong Agricultural University, Taian, 271018 China; Sino-Africa Joint Research Center, Chinese Academy of Sciences, Wuhan, 430074 China

**Keywords:** CRISPR/Cas9, Database, Genome editing, PAM, *Vitis vinifera*

## Abstract

**Background:**

CRISPR/Cas9 has been recently demonstrated as an effective and popular genome editing tool for modifying genomes of humans, animals, microorganisms, and plants. Success of such genome editing is highly dependent on the availability of suitable target sites in the genomes to be edited. Many specific target sites for CRISPR/Cas9 have been computationally identified for several annual model and crop species, but such sites have not been reported for perennial, woody fruit species. In this study, we identified and characterized five types of CRISPR/Cas9 target sites in the widely cultivated grape species *Vitis vinifera* and developed a user-friendly database for editing grape genomes in the future.

**Results:**

A total of 35,767,960 potential CRISPR/Cas9 target sites were identified from grape genomes in this study. Among them, 22,597,817 target sites were mapped to specific genomic locations and 7,269,788 were found to be highly specific. Protospacers and PAMs were found to distribute uniformly and abundantly in the grape genomes. They were present in all the structural elements of genes with the coding region having the highest abundance. Five PAM types, TGG, AGG, GGG, CGG and NGG, were observed. With the exception of the NGG type, they were abundantly present in the grape genomes. Synteny analysis of similar genes revealed that the synteny of protospacers matched the synteny of homologous genes. A user-friendly database containing protospacers and detailed information of the sites was developed and is available for public use at the Grape-CRISPR website (http://biodb.sdau.edu.cn/gc/index.html).

**Conclusion:**

Grape genomes harbour millions of potential CRISPR/Cas9 target sites. These sites are widely distributed among and within chromosomes with predominant abundance in the coding regions of genes. We developed a publicly-accessible Grape-CRISPR database for facilitating the use of the CRISPR/Cas9 system as a genome editing tool for functional studies and molecular breeding of grapes. Among other functions, the database allows users to identify and select multi-protospacers for editing similar sequences in grape genomes simultaneously.

**Electronic supplementary material:**

The online version of this article (doi:10.1186/s12870-016-0787-3) contains supplementary material, which is available to authorized users.

## Background

CRISPR (clustered regularly-interspaced short palindromic repeats)/Cas (CRISPR associated protein) has recently emerged as an effective genome editing system for modifying genes in a wide range of organisms, including humans, animals, bacteria and plants [1–3]. The system has three types: I, II, and III, and maintains high specificity through canonical Watson-Crick base pairing of guide RNAs to the target sites. Type II uses Cas9 nucleases and is the most useful system demonstrated so far [1] due to the unique properties of the enzymes. A Cas9 nuclease can be guided by CRISPR to a targeted protospacer region, located at the upstream of a protospacer-adjacent motif (PAM). Then, the Cas9 nuclease can induce precise cleavages at the endogenous genomic locus resulting in DNA deletion and other changes at the locus [4]. In addition, the Cas9 nuclease can be converted into a nicking enzyme to facilitate homology-directed repair with mutagenic activity [4]. These properties of CRISPR/Cas9 make the system a valuable and versatile tool for many research applications [5, 6].

Successful examples of using the CRISPR/Cas9 system for various genome-editing purposes are accumulating at a fast pace. The system was used to introduce precise mutations into the genomes of *Streptococcus pneumoniae* and *Escherichia coli* in 2013 [2], which demonstrated the effectiveness and versatility of the technique for bacterial genome engineering. Subsequently, the CRISPR/Cas9 system of prokaryotic *Streptococcus pyogenes* was employed as programmable RNA-guided endonucleases to cleave DNA in a targeted manner for genome editing in human and mouse cells [3, 4]. Now, various tool kits with vectors carrying pGreen or pCAMBIA backbones, which can facilitate transient or stable expression of the CRISPR/Cas9 system, have been developed for multiplex gene editing in plants [5]. Genes from many plant species, including *Arabidopsis thaliana*, *Triticum aestivum*, *Lycopersicon esculentum*, *Citrus sinensis* and *Nicotiana*, have been successfully edited by using the CRISPR/Cas9 system [7–11]. Furthermore, several databases and web tools have been established to facilitate related studies [12–14].

Grape is one of the most important fruit crops in the world, and its draft genome sequence was first released in 2007 by assembling eight-fold shotgun sequences and later improved by increasing the coverage to 12-fold [15, 16]. Because of the economic importance of grapes, it is conceivable that the CRISPR/Cas9 system will soon be adopted for editing grape genomes for various research and applied purposes. To accelerate adoption of this genome-editing technology in grapes, we analyzed grape genome sequences and identified millions of potential protospacers and PAMs for CRISPR/Cas9-based genome editing. In addition, we developed a user-friendly grape CRISPR database and made it available for public use.

## Results

### Genomic distribution of protospacers and PAMs

**Fig. 1:**
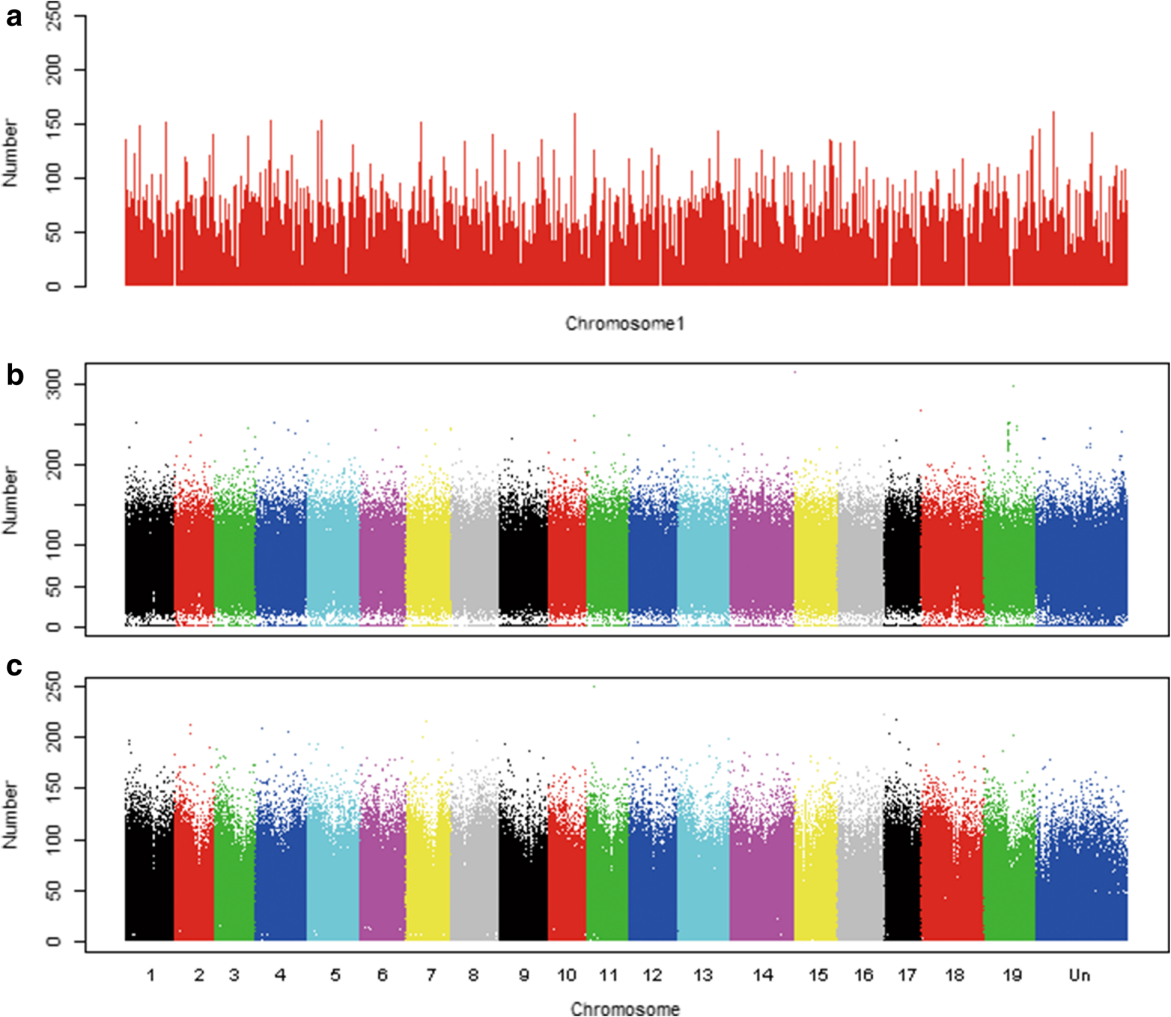
Distribution patterns of protospacers in the grape genome. **a** Schematic illustration of protospacers in chromosome 1. Bar width represents one Kb-long genomic sequence, and the bar height stands for protospacer abundance in the one Kb-long sequence. **b** Distribution patterns of protospacers in the 19 grape chromosomes and the unassigned chromosomal regions (ChrUn). Each point on the figure represents relative abundance of protospacers in the given 1 Kb region. **c** Distribution of unique protospacers on the 19 grape chromosomes and the unassigned chromosomal regions (ChrUn)

A total of 35,767,960 protospacer/PAMs were detected in the draft grape genome, and 63.18 % of them (22,597,817) were present at specific genomic locations. On average, the number of protospacer/PAMs in the genome was 73.57/Kb overall with 46.48/Kb site-specific (Table 1). These protospacers appeared evenly distributed among and within chromosomes. As an illustration (Fig. 1a), the protospacers on chromosome 1 were more or less evenly distributed, although the abundance of the protospacer/PAMs ranged from 0 to 252/Kb for the chromosome. The other chromosomes had similar distribution patterns (Additional file 1). Depending upon the length of a chromosome, the number of protospacers varied among the 20 chromosomes (19 known linkage groups and 1 with random markers unmapped). The total protospacers ranged from 1, 303, 573 in chr17 to 2, 978, 796 in chrUn, and unique protospacers (the protospacers which appeared only once in the whole genome) ranged from 835, 838 in chr10 to 1, 495, 033 in the chr14 (Additional file 2). When the numbers of total and unique sites were compared, there were no significant differences in their distribution among the different chromosomes (Figs. 1b and c, respectively), suggesting that each chromosome had a similar level of protospacer/PAM abundance and the overall distribution was relatively uniform. It was noted that the abundance of specific protospacers in both arms of the chromosomes were higher than in the central regions (Fig. 1c). In addition, there was a significantly positive correlative relationship between the numbers of total and unique protospacers. Furthermore, about one third of the unique protospacers (7,269,788) were highly specific.

**Table 1:** Numbers of cleavage sites and their distribution patterns in the grape genome

	Overall (no.)	Relative abundance (no./KB)	Average no./genomic region	Site-specific	Relative abundance (no./KB)	Average no./genomic region
Genome	35,767,960	73.57		22,597,817	46.48	
Intergenic	21,895,244	69.25		11,985,746	37.91	
Gene	13,872,716	81.59	526.56	10,612,071	62.41	402.80
Exon	3,802,381	97.36	23.46	3,209,457	82.18	19.81
Intron	9,260,692	75.98	68.24	6,712,250	55.07	49.46
UTR	809,643	89.01	22.61	690,364	75.90	19.28

### Composition of PAMs

**Fig. 2:**
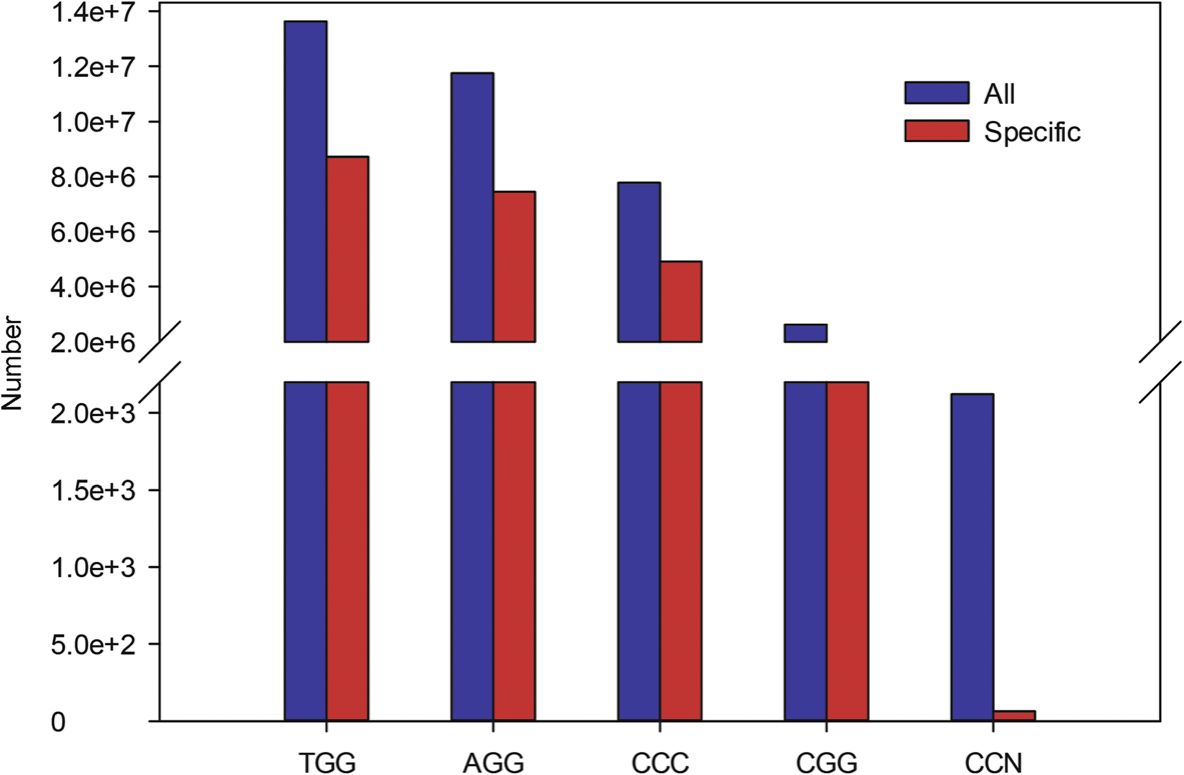
The type and number of PAMs identified in the grape genome

Five PAM types were observed, including TGG, AGG, GGG, CGG and NGG. The NGG type was observed at a very low frequency (0.0029 %) and likely resulted from low-quality sequences. The other four PAM types were present on all of the chromosomes. TGG was the most abundant one, followed by AGG, GGG and CGG, and they accounted for 38.10, 32.75, 21.74 and 7.40 % of the total PAMs, respectively (Fig. 2 and Additional file 1). As far as PAM types are concerned, there was no significant difference between the total and unique PAMs through the whole grape genome (Fig. 2).

### Cleaving sites in different genomic regions

We surveyed the distribution of cleavage sites in various regions of the grape genomes (Table 1). The number of cleavage sites in the intergenic region was almost 2-fold more than that in the genic region (21,895,244 vs. 13,872,716). However, the relative abundance of cleavage sites in genic regions was higher than that in intergenic regions for both overall (81.59/Kb vs. 69.25/Kb) and unique (62.41/Kb vs. 37.91/Kb) PAMs. In genic regions, the number of cleavage sites in introns was more than that in exons, which had about the same number of cleavage sites as were found in the UTR regions. Further, the abundance of cleavage sites in exons and UTRs was higher than that in introns. On average, about 526.56 total cleavage sites and 402.80 unique ones were present in a gene.

### Synteny analysis of similar genes and multi-protospacers

**Fig. 3:**
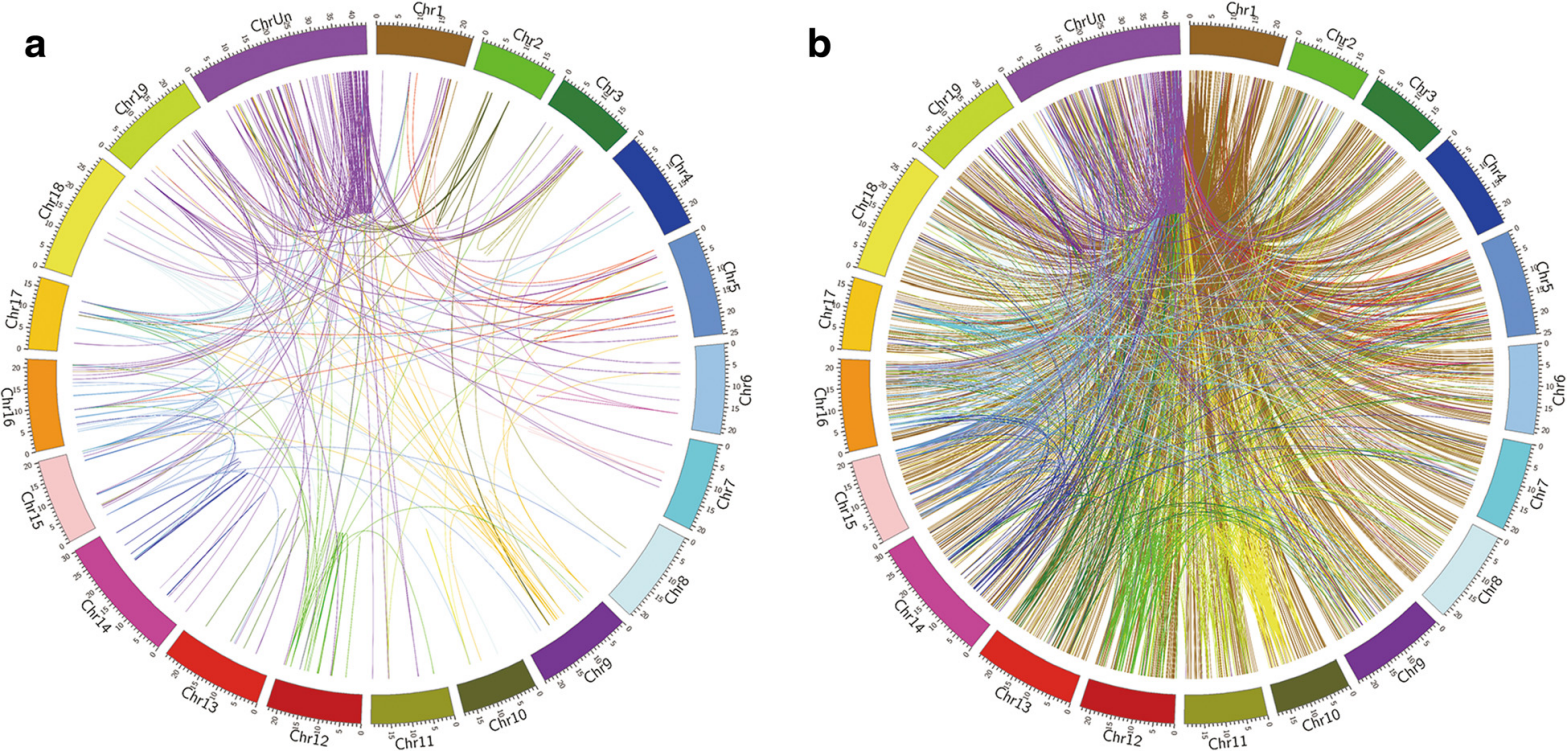
Comparative analysis of the relationships of homologous genes and their multi-protospacers in the grape genome. **a** Relationships of 141 highly homologous genes. Two genes that were linked by a line shared sequence segments with high homology; **b** Synteny analysis of multi-protospacers. The same protospacers were connected by lines

Most genes had both unique and non-unique protospacers with the exception of 141 genes (about 0.5 %) which contained only non-unique protospacers. These 141 genes were scattered in all the chromosomes containing a total of 4294 protospacers, and had an average of 30.45 protospacers per gene. Synteny analysis of similar genes revealed that the synteny of protospacers matched the synteny of homologous genes. Each individual gene might have several protospacers, and some of these protospacers could be found in all of the individual gene members of the same group or family (Fig. 3a and b).

### Grape-CRISPR database

To facilitate identification of suitable genomic target sites for editing grape genomes using the CRISPR/Cas9 system, we developed a searchable database (named as Grape-CRISPR database). The database contains two main sections: Search and Design. In the Search section, users can identify appropriate protospacer and PAM sites of a gene by providing certain inquiry information such as locus location, gene ID or Pfam ID. The database will provide an overall score of 1–3 for each spacer on the basis of its GC content and PAM (NGG or GGNGG) type. It will also indicate if a protospacer of interest can be easily incorporated into an expression vector with U6 or T7 promoter. If a spacer is non-unique, a circus map will be provided to show its relationship with others. The Design section is for protospacer design. Users can detect and design protospacers and PAMs in the sequences of interest by using the Perl scripts provided.

## Discussion

Protospacers and PAMs were abundantly present in the grape genomes. These protospacers and PAMs were more or less uniformly distributed among chromosomes and chromosomal regions. The abundant presence and uniform distribution pattern of these potential target sites provide the possibility for editing most of the grape genomic regions by using the CRISPR/Cas9 system. The fact that most genes contain many specific/unique protospacers allows grape researchers to edit a gene of interest with multiple choices of target sites and great specificity. The uniform distribution pattern of protospacers and PAMs among and within chromosomes suggests that these target sites were apparently not associated with any specific properties of the grape chromosomes. However, the relative abundance of cleavage sites in the genic regions, in coding regions in particular, were higher than that in the intergenic regions.

Among the five PAM types, TGG and AGG types were the most abundant. However, there was no significant statistical difference in their frequencies of occurrence among all the PAM types except for the NGG type which was much lower. The type of NGG was a special one which included an ambiguous base pair. These NGG PAMs were mainly present in regions with a low quality of genomic sequence information, possibly due to the presence of repetitive sequences. In practice, one can use any of the TGG, AGG, GGG, and CGG target sites, but not the NGG type, for genome-editing in grapes. Our synteny analysis showed that multi-protospacers had synteny with their homologous genes. Based on sequence similarity, one could use a universal protospacer to guide the CRISPR/Cas9 system simultaneously to edit several genomic sites at one time. This will be especially useful for modifying homologous genes or family genes of interest. Because grape is a highly heterozygous species and SNPs are abundant in the genomes, it would be prudent and useful to re-sequence potential target sites to confirm them and avoid potential mismatches due to the presence of a SNP(s) between the reference genome and the grape variety or species of interest.

One of the important outcomes from this study was the development of a Grape-CRISPR database. Compared with other similar databases [12–14], the Grape-CRISPR database was developed on the basis of a thorough genome-wide analysis of grape genome sequences. We provide annotation, gene ID and PFAM number information for specific protospacers, which makes the database more informative to users. This database also contains considerably more data than other similar databases and, more importantly, we provide custom Perl scripts to scan and filter the database. By doing so, the database will allow users to explore various options and to extract relevant information from it.

## Conclusion

Grape genomes contain a large number of PAM sites and protospacers for potential genome editing by use of the CRISPR /Cas9 system. These sites are widely and more or less evenly distributed among and within chromosomes. The presence of many potential target sites in the grape genomes, and the relatively higher abundance of cleavage sites in the genic regions than in the intergenic regions, provide an encouraging future perspective to edit grape genomes by use of the CRISPR/Cas9 system. In addition to charactering various properties of protospacer and PAM sites, we developed a Grape-CRISPR database for public use.

## Methods

The grape genome sequence and annotation information (*Vitis vinifera* 12X) used in this study were downloaded from the phytozome at http://phytozome.jgi.doe.gov/pz/portal.html.

### Identification and distribution of PAM sites and protospacers

In previous studies, it was found that NGG (or CCN on the complementary strand) sequences are sufficient for targeting [2]. Therefore, only NGG (CCN) was considered as a potential PAM site, and the protospacer length was set as 20 bp in this study. The protospacers and PAM sites were detected by a Perl script that we wrote. All possible sites were taken into consideration. In the case that a sequence contains poly G (Gn) or poly C (Cn), the PAM number will be counted as n-1. All the protospacers were assessed on the basis of their 20 bp-long sequences, and the protospacers which appeared only one time were identified and noted as “specific protospacers”. Then, a further tolerance test was carried out to identify “highly specific” protospacers. The test allows at most two mismatches in the protospacers and the last three bases must have high fidelity. This test was done by using BLASTN.

The average abundance of all PAMs and specific PAMs per 1 Kb-long sequence were compared for 20 chromosomes (19 known linkage groups and 1 with random markers unmapped), and the correlation between the abundance of all and specific PAMs was determined.

The NGG (CCN) PAMs were classified into five types in this study: AGG (CCT), TGG (CCA), GGG (CCC), CGG (CCG), and NGG (CCN) where the N is an ambiguous base pair. If a PAM was associated with a specific protospacer, then the PAM was considered and counted as a specific one.

### Identification of Cas9 cleavage sites

Previous studies showed that the Cas9 enzyme cleaves the target sequence at the site 3 base pairs upstream of the PAM, but on the complementary strand there may be several cleavage sites from 3 to 8 base pairs upstream of the PAM [1]. In this study, we focused on the cleavage sites 3 base pairs upstream of the PAMs. The distribution patterns of these cleavage sites in the genome and intergenic, gene, exon, intron and UTR elements were determined on the basis of available annotation information.

### Synteny assessment of multiple protospacers and corresponding genes

There are genes which contain multiple protospacers and therefore cannot be effectively edited individually. However, if these genes are similar in their sequences and functions, they might be edited as a group or gene family. We used similar genes as query sequences and blasted them against a local CDS database to determine the similarity of these genes. For those genes which shared segment similarities higher than 80 %, we propose that they might be good candidates for group editing. The genomic locations of the protospacers of these genes were also located. The synteny results were used to analyze the possibility of editing similar genes together.

### Database architecture and web interface

All the data obtained in this study are stored in the Grape-CRISPR Database (http://biodb.sdau.edu.cn/gc/index.html). The database contains interrelated relational databases implemented through MySQL and a web interface running function on an Apache web server implemented through HTML and PHP (Additional file 3). The database is based on a Linux sever and can be freely accessed through the internet. It contains information of CRISPR /Cas9 site properties such as gene IDs, genome loci, protospacers, GC content, and promoter applicability. The database also contains relationship information among all the Cas9 sites, with a PFAM annotation database containing the candidate genes of each PFAM model. The interface was written using HTML and CSS. The user inquiries were uploaded to the system and processed by PHP and MYSQL or Perl scripts.

### Ethics approval and consent to participate

Not applicable

### Consent for publication

Not applicable

### Availability of data and materials

The protospacers, annotation information and all other detail data in this article all can be searched and browsed from the Grape-Crispr database (http://biodb.sdau.edu.cn/gc/).

## Additional files

Additional file 1: Distribution patterns of CRISPR/Cas9 sites on individual grape chromosome. (TIFF 2645 kb) Additional file 2: Numbers and compositions of the observed PAMs on individual chromosomes. (XLS 33 kb) Additional file 3: Schematic illustration of the “Search” and “Design” components in the Grape-CRISPR database. (TIF 3164 kb)

## Abbreviations

Cas: CRISPR -associated protein
CRISPR: clustered regularly interspaced short palindromic repeats
PAM: protospacer-adjacent motif
