# Supplementary figures and images for: Identification of genomic sites for CRISPR/Cas9-based genome editing in the Vitis vinifera genome
Source: BMC Plant Biol. 2016 Apr 21;16:96. doi: 10.1186/s12870-016-0787-3 (PMC4839089; doi:10.1186/s12870-016-0787-3)

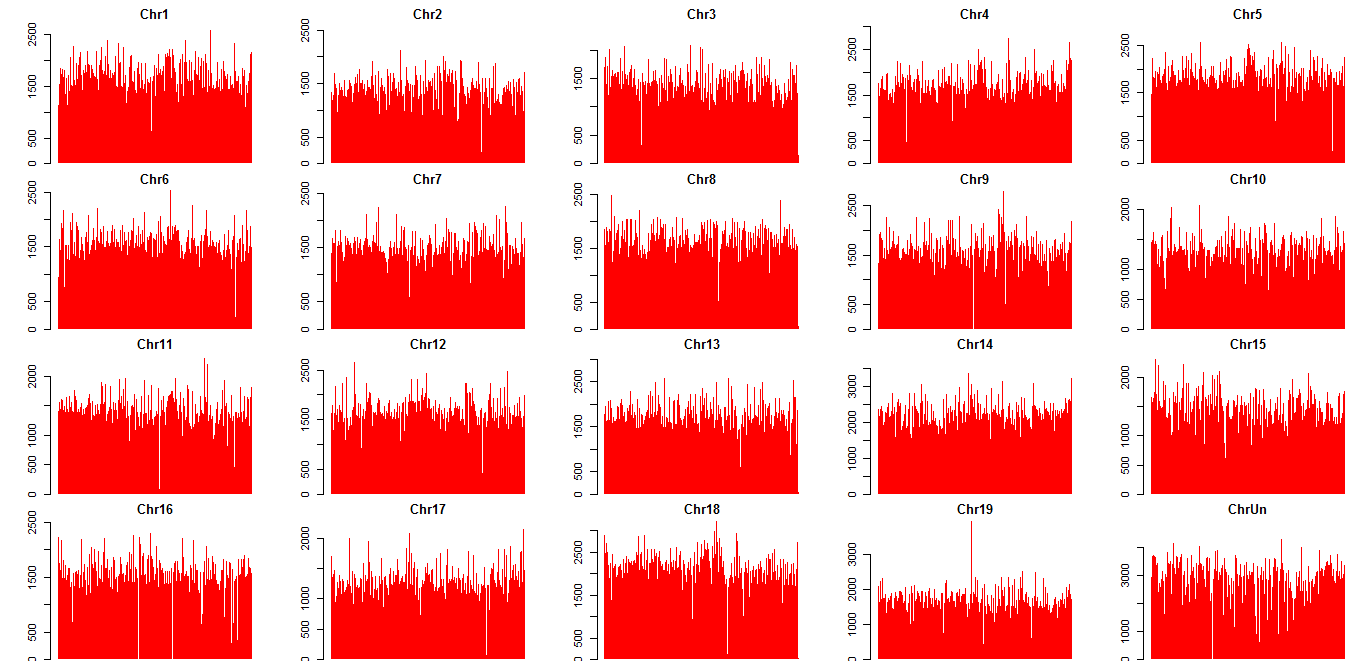

Supplement: Additional file 1: — Distribution patterns of CRISPR/Cas9 sites on individual grape chromosome. (TIFF 2645 kb) [file 12870_2016_787_MOESM1_ESM.tiff]

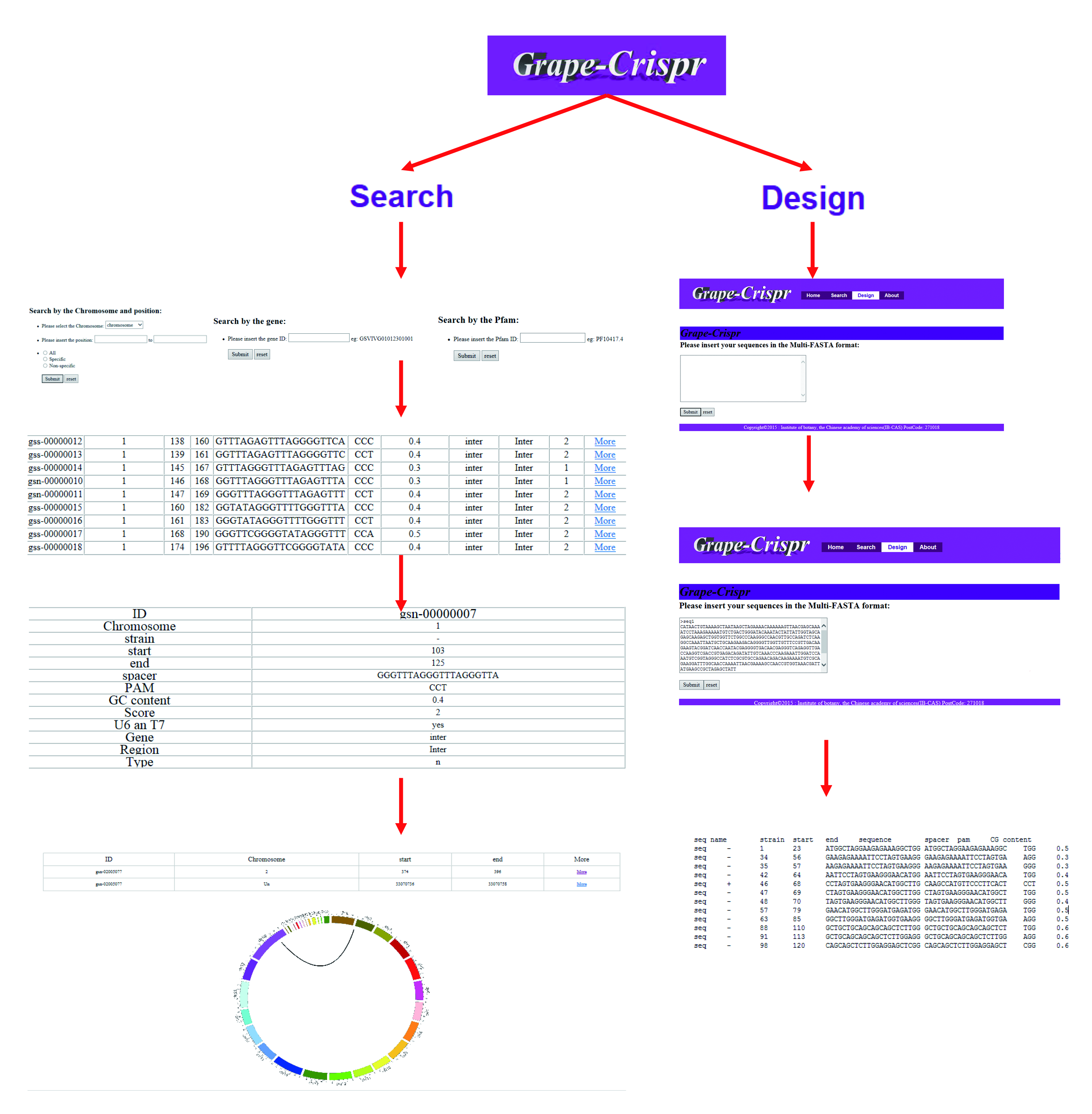

Supplement: Additional file 3: — Schematic illustration of the “Search” and “Design” components in the Grape-CRISPR database. (TIF 3164 kb) [file 12870_2016_787_MOESM3_ESM.tif]
